# Pull or Push? Octopuses Solve a Puzzle Problem

**DOI:** 10.1371/journal.pone.0152048

**Published:** 2016-03-22

**Authors:** Jonas N. Richter, Binyamin Hochner, Michael J. Kuba

**Affiliations:** Department of Neurobiology, Alexander Silberman Institute of Life Sciences, Hebrew University of Jerusalem, Givat Ram, Israel; University of Sussex, UNITED KINGDOM

## Abstract

Octopuses have large brains and exhibit complex behaviors, but relatively little is known about their cognitive abilities. Here we present data from a five-level learning and problem-solving experiment. Seven octopuses (*Octopus vulgaris*) were first trained to open an L shaped container to retrieve food (level 0). After learning the initial task all animals followed the same experimental protocol, first they had to retrieve this L shaped container, presented at the same orientation, through a tight fitting hole in a clear Perspex partition (level 1). This required the octopuses to perform both pull and release or push actions. After reaching criterion the animals advanced to the next stage of the test, which would be a different consistent orientation of the object (level 2) at the start of the trial, an opaque barrier (level 3) or a random orientation of the object (level 4). All octopuses were successful in reaching criterion in all levels of the task. At the onset of each new level the performance of the animals dropped, shown as an increase in working times. However, they adapted quickly so that overall working times were not significantly different between levels. Our findings indicate that octopuses show behavioral flexibility by quickly adapting to a change in a task. This can be compared to tests in other species where subjects had to conduct actions comprised of a set of motor actions that cannot be understood by a simple learning rule alone.

## Introduction

Octopuses represent an important and interesting model for comparative cognition. They have a unique morphology, intricate behavior and the most complex central nervous system among all invertebrates. Animal cognition has advanced greatly in recent years and new findings in both vertebrates and invertebrates elicited the interest in the evolution of cognition. Still, most research focuses on primates, birds, and to some extent insects, and while cephalopods show great potential, experimental data on cognition in octopus is scarce.

Innovative behavior or problem solving means finding a solution to a novel problem or a novel solution to an old one [1]. Theory predicts that such cognitive abilities are favored in species that exploit diverse food sources, have complex social structure, inhabit environments with highly unpredictable resources and undergo relatively long developmental stages [2, 3]. Octopuses are reported to be exploratory and attracted by novel objects [4], opportunistic feeders [5, 6], forage large territories [7, 8] and migrate vertically and horizontally [5]. While octopuses don’t have rich social structure or particularly long developmental stages, their ecology and behavior at the adult benthic stage is very variable and complex, representing the dynamic near shore environments in which *O*. *vulgari*s is found [9, 10].

Innovation has been correlated to cephalization, i.e. cortex and pallium size, while this could be shown in birds and monkeys [11], but, cognitive traits are shown in insects not possessing these brain areas [12–14]. The octopus brain with about 140 million neurons [15, 16] is large and complex compared to other invertebrate brains. Their brain-weight-to-body-weight ratio is comparable to that of vertebrates [17] and the vertical lobe, which is involved in long term memory [18], shares some functional features with the vertebrate hippocampus and the insect mushroom bodies [19–21]. In many behavioral studies octopuses show learning and memory abilities [22, 23] and readily solve discrimination tasks [24–27].

However, despite the excellent learning abilities of octopuses, only a few cognitive abilities have been investigated, e.g. play behavior [28], navigation [8, 29, 30] and detour task solving in a maze [31, 32]. Some reasons for this scarcity are technical difficulties, the lack of standardized and practical training protocols, as well as technical devices and apparatuses. The special morphology of octopuses, their eight highly flexible arms and soft body, makes it difficult to restrain the animals for experimental set-ups. Classic cognition experiments like *problem solving* often incorporated applications (e.g. the *trap-tube problem* [33, 34]), which are difficult to test in octopuses because of their flexible arms. A lever-pulling experiment, similar to a skinner-box, proved to be inapplicable to octopuses as they failed to learn the task [35]. Fiorito et al. [36] overcame some of the difficulties using a jar-opening task, which exploited the animal’s natural exploration and manipulation instincts. In his experiments, *Octopus vulgaris* had to solve a novel problem in form of a glass jar, which had a food item in it and was closed by a rubber plug. Fiorito et al. [36] (also see [37, 38]) showed a simple and successful problem solving experiment, in which the task remained constant throughout the entire experiment. However, several unanswered questions remain—did the animals solve the task by stimulus-response association or by trial-and-error and whether octopuses solve more complex problems [39]?

In order to test flexible behavior and problem-solving strategies in octopuses, we developed a series of experiments consisting of a learning and a systematically changing problem solving task. All seven subjects solved the two basic tasks, to open the L-shaped container in level 0 and to pull it through the separator hole in the subsequent puzzle-task levels, and thus showed behavioral flexibility.

## Materials and Methods

### Subjects and holding

Subjects were seven wild-caught *Octopus vulgaris* (4 females, 3 males; between 250–500g bodyweight) collected by fishermen from the Israeli coast of the Mediterranean Sea. The animals were housed individually in a semi closed system of glass aquaria (100cm x 40cm x 40cm) and visually shielded from their conspecifics. Using a water chiller, temperature was held constant at about 24°C. According to the guidelines for the EU Directive 2010/63/EU for cephalopod welfare [40] aquaria were enriched with clay-pot dens, gravel, rocks and green algae (*Caulerpa prolifera*). Animals were fed every other day with either shrimps or pieces of fish. All animals acclimatized for at least 14 days in the holding tanks before experiments started. Animals were monitored daily by either the authors of the study or by students from the local college for aquaculture. Animals were preselected for both adaptation to human care and general health. This was judged by checking if the animals were regularly and readily feeding and by inspection for skin lesions, visible parasites and general health status. During the experiments all animals were fed a regular diet of dead fish and crustaceans.

### Experiments

**Fig 1:**
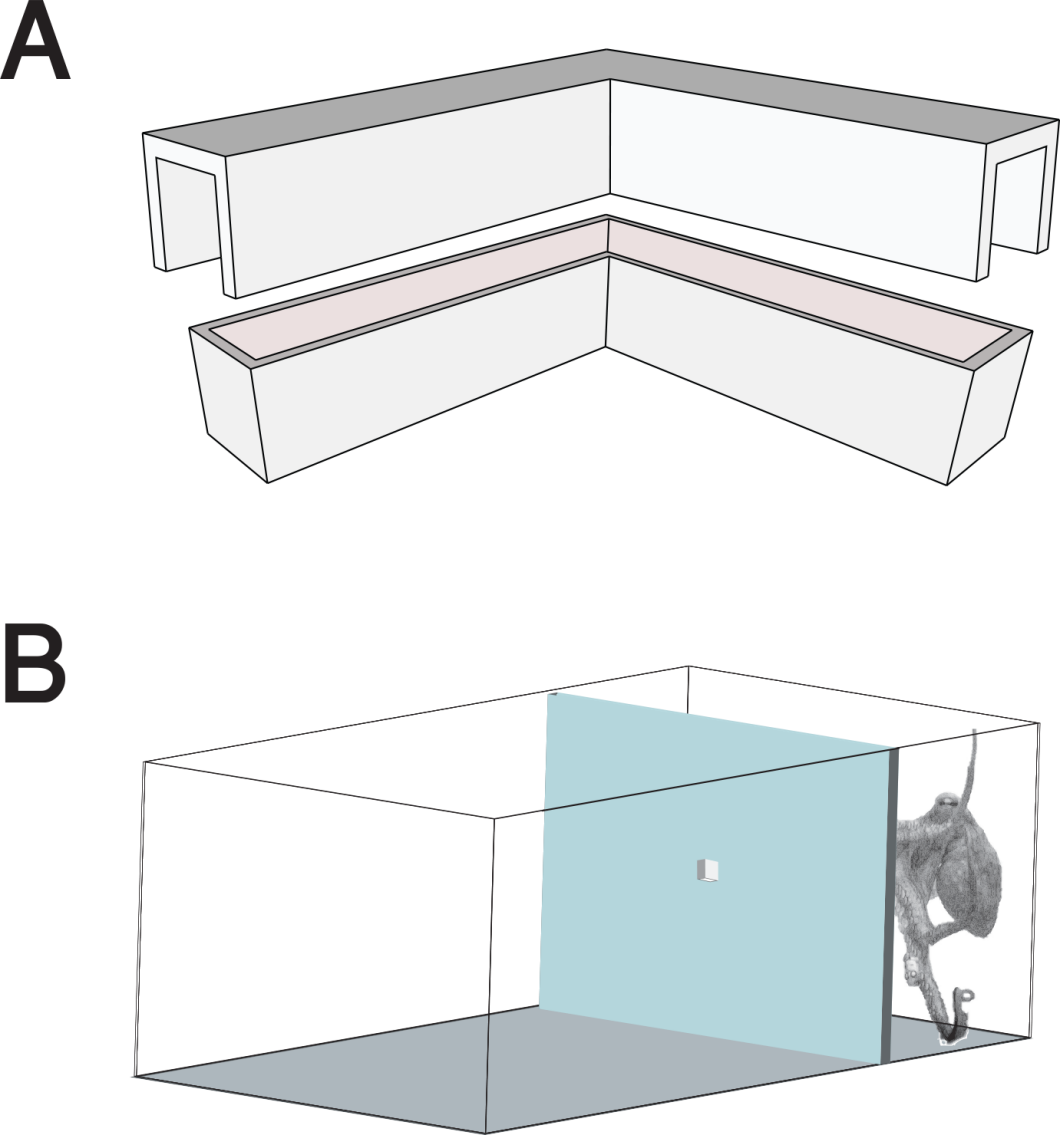
Experiment set-up. (A) Two halves of the L-shaped container. Length of one arm 6 cm, width 1.4 cm, height 2 cm. (B) Schematic set up of the experiment tank with an opaque Perspex separator (light blue) and the stimulus hole in the center. Subjects were placed in the smaller compartment 30 min before the onset of the experiments.

The unique morphology of octopuses can present a challenge for researchers and the experimental design. Octopuses are typically very curious they pounce and manipulate novel moving objects. They often forcefully manipulate lighter and moving objects or experimental apparatuses until they break. The presence of observers can influence the animals behavior, handling of the animals can constitute an immense stress factor, especially since contact with an object (e.g. a net or the researchers arm) can lead to a “tug of war” over it [39]. Motivation of the animals can wane quickly after an object was explored and yielded no food reinforcement for the octopus [4]. The aim of the current study was to use the natural behavior of octopuses to develop a complex problem-solving task with several levels, which built upon each other. To that end we restricted each animal to a compartment of the tank (30 min before the start), which was closed off by a Perspex separator (Fig 1B). This restrained the animal to an approximately 10×30×30 cm space, which in turn eased stimulus presentation and trial onset and limited the handling of the animal. Between trials there were at least ten minute inter-trial intervals. Each day ten experimental trials were conducted. Experiments were carried out in the home tank of each individual animal, which reduced time for adaptation to an experimental setting. After the experiments the partitions were removed and the animals had again access to their entire home tank. Such temporary restraining has been successfully done in a previous study [41] and did not have any measurable effect on the wellbeing of the animal. The following tasks were presented to the subjects:

Container-opening task (level 0); a training level to form an association between the L-shaped container (Fig 1A) and a food reward. A piece of shrimp was placed inside the L-shaped container and presented to the animal, which then had to open the container within a 5 minute time period. Success criterion was an opened container.

Puzzle task (level 1–4); individual subjects were restricted to one part of the tank and had to manipulate the L-shaped container through a tightly fit hole in the separator in order to get the food reward. The container was presented to them with one leg of the container put in the hole and the other leg pointing upwards. In order to manipulate the container through the hole, it had to be accurately leveraged around the corner of the container, as the tightly fit hole left no room to manipulate the container through the hole otherwise. The tight fit of the container made it impossible to open the container while in the hole.

The task was altered for each of the four levels: In level 1, the first stage of the puzzle task, the separator was transparent and the orientation of the container was upwards (S1 Video). In level 2 the transparent Perspex separator was exchanged for an opaque separator, but the container-orientation remained upwards. The opaque separator was also used for the next two levels. In level 3 the orientation of the container was reversed, the outside leg pointed down. In level 4 the orientation of the container was randomized, using all four possible orientations (S2 Video). Randomization was done according to Fellows [42] for each animal, with the first trial set to an upward orientation for motivational purposes.

The stimulus presentation through a small hole in the separator wall reduced further contact and presented a cue for trial onset. Once an item is grabbed, octopuses hardly let go and forcefully pull on it [39]. In order to get to the food-containing object, however, the animals needed to learn to overcome their initial instinctive behavior and not pull, but lever the tight fitted L-shaped box through the hole.

We decided to apply a strict criterion to ensure that each animal passed to the next level with equal mastery of the task. The success criteria for level 0–2 were three consecutive experiment days of 80% successful trials. We assumed that these levels were the most difficult, and that therefore it was important to establish equal proficiency in all subjects before proceeding to levels 3 and 4. After the initial capacity for box manipulation had been established, criterion for testing in levels 3 and 4 was one experiment day with 80% successful trials. Each experiment day consisted of 10 trials, except level 4 with 12 trials to account for equal distribution of the four orientations. Trials were marked successful in level 1 to 4 when the corner piece was pulled to the other side of the separator. The trial was reset, when animals turned the container to different orientations, dropped the container, or after 5 minutes of no success.

### Analysis

The sessions were recorded with a digital video camera (SONY Handycam HDR-XR550; Tokyo, Japan) and later analyzed with The Observer XT 10 (Noldus Information Technologies, Wageningen, Netherlands). Further data analysis was done with SPSS 19 (IBM Software; Armonk, New York, USA) and Microsoft Excel 2011 for Mac OS (Redmond, Washington, USA).

Over all 1469 trials were analyzed, of which 240 (16.3%) were marked as fails and 1229 were used for further analysis. Mean (± SD) number of experiment days for all levels and animals was 20 (± 6.9) with a minimum of 11 days by experiment design (S1 Data).

To reduce high variance of trial durations, which was caused by animals not behaving during the experiments, only behaviors scored as “working” were used for time analysis. Working times consisted of durations that were subjectively scored while the animal showed active interaction with the container. The shape of the distribution of durations was examined and found to be similar to the overall trial time. To calculate learning effects over the course of the experiment, trial bins were created for level 1 to 4. Trials for level 1 and level 2 were divided into three equally-sized trial bins per level and animal. Since the criterion for the test situations in level 3 and level 4 was only one successful experiment day, each level formed one trial bin.

To analyze performance between different trial bins or experiment days a Wilcoxon signed rank test was used. The regression of working times within level 1 and 2 was analyzed with Kendall’s tau. To test for differences in the orientation of the container during level 4, a Kruskal-Wallis H test was employed.

### Ethical Statement

All experiments took place before the passing of the EU directive 2010/63/EU on the protection of laboratory animals. However, due to the involvement of the authors in the process of developing the guidelines regarding the use of cephalopods as laboratory animals, all experimentation and housing guidelines of the EU directive were followed as if the regulations were in place. All experiments conducted were non-invasive behavioral experiments and no negative reinforcement was used at any time (S1 and S2 Texts). Animals were kept in a flow-through system and therefore always-in natural water conditions. After completing the experiments, animals were released back into the wild. The animal protection law in Israel does not cover invertebrates, thus no evaluation by an ethics commission was possible.

## Results

### Level 0–2: Learning and problem solving performances

**Fig 2:**
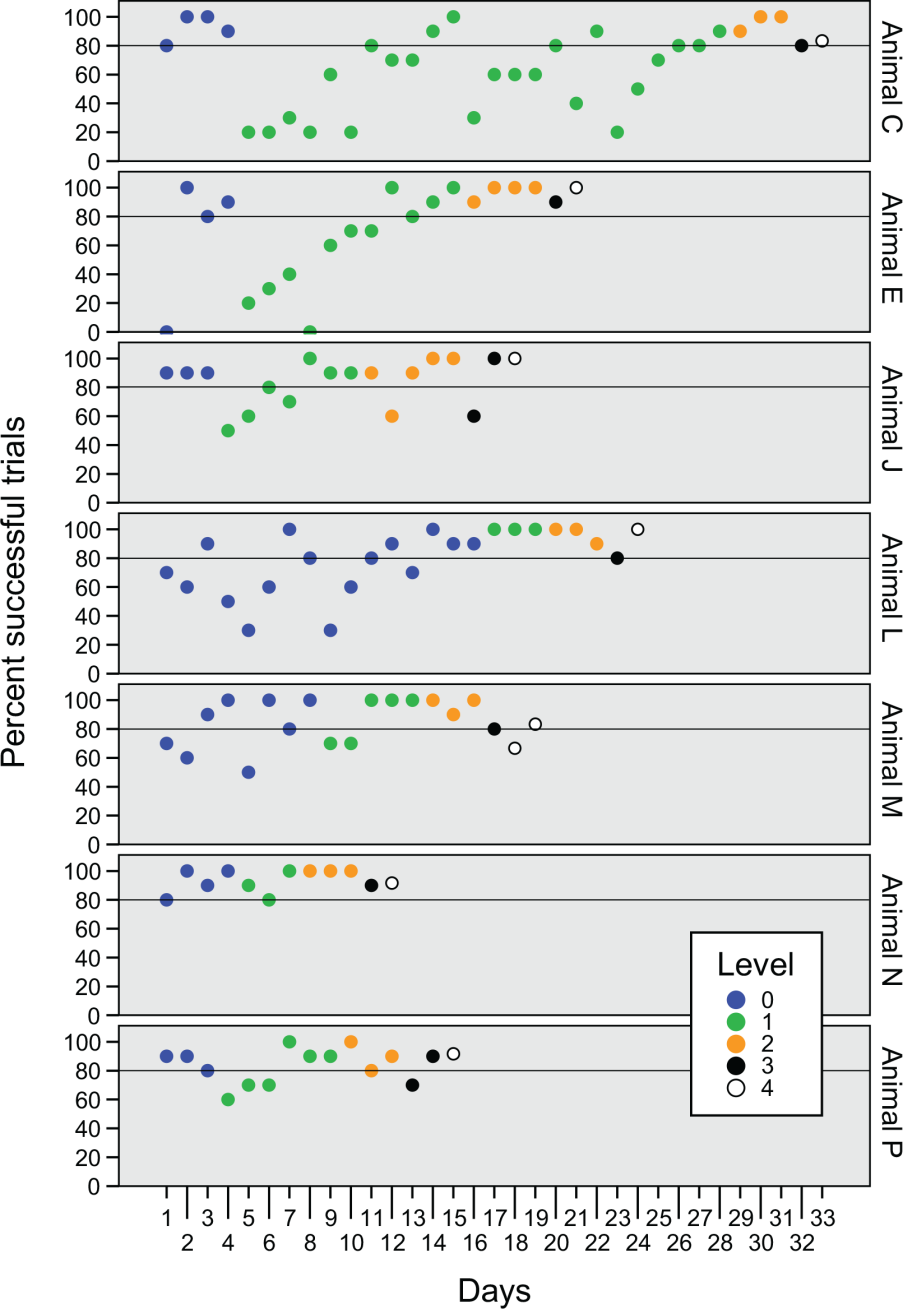
Success rates for individual animals over all experiment days. Blue circles: Pre-training level 0; food association with L-shaped container. Green circles: training level 1; introduction of a transparent separator. Orange circles: level 2; introduction of an opaque separator. Black circles: test situation level 3; downward oriented container. White circles: level 4; container randomly oriented in 4 directions. Black line marks 80% success criterion.

All animals learned to solve the task in level 0 and showed a relatively short (1.94 ±1.81 sec) mean contact latency (the time between the insertion of the container into the water and first contact). Animals showed inter-individual differences in the amount of time it took them to successfully accomplish the task in level 0 and the subsequent task in level 1 (Fig 2, blue and green circles). For example, Animal C passed level 0 after four experiment days and level 1 after 24 days. On the other hand, Animal L passed level 0 after 16 experiment days and level 1 after seven days. After level 1 was passed, all animals had a significant decrease in trial numbers to reach success criteria in level 2 (Wilcoxon signed-rank test; z = -2.032, p = 0.042; see Fig 2, yellow circles).

**Fig 3:**
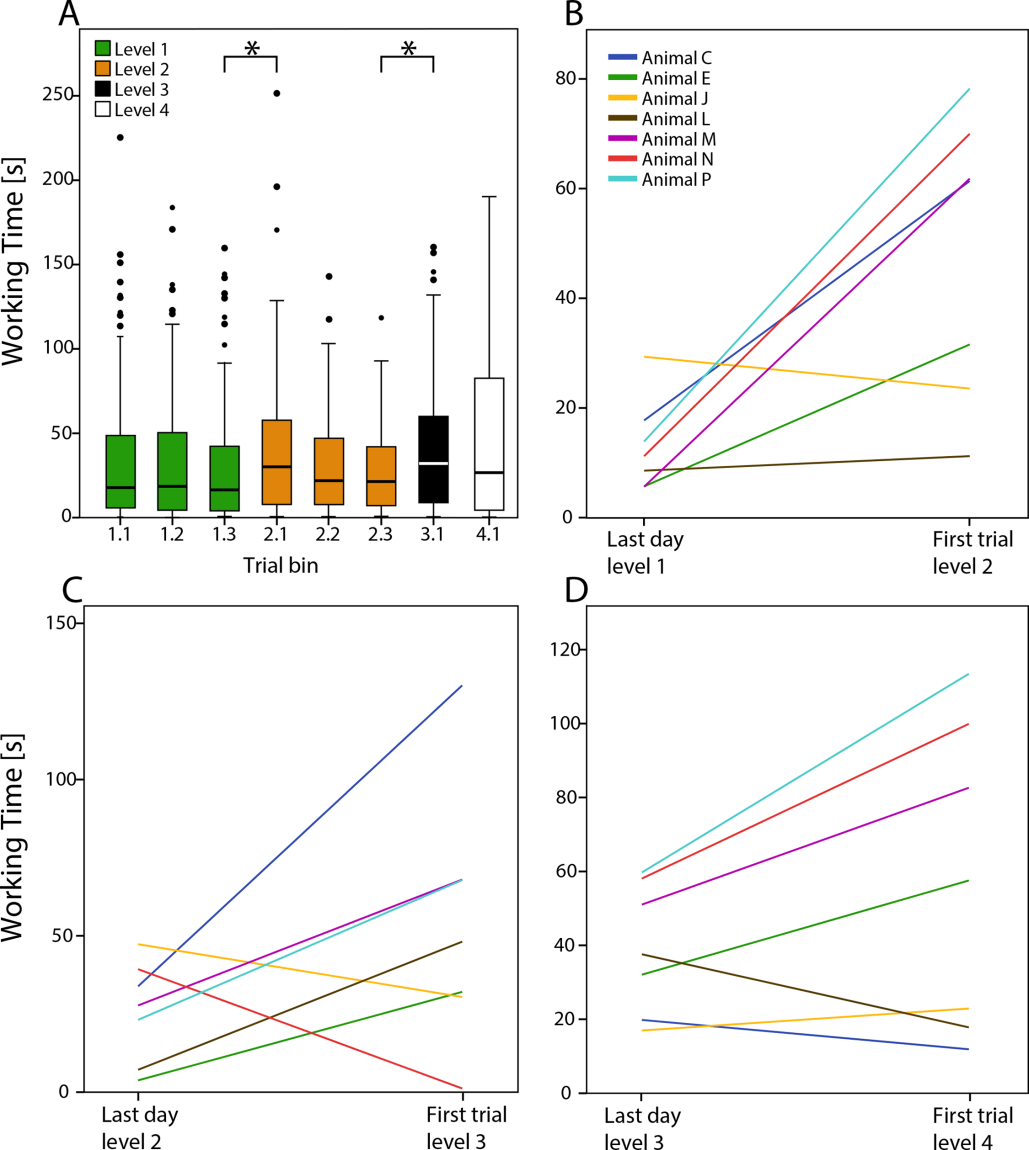
**Boxplot of working times over trial bins for grouped animals** (A). Central line indicates median; boxes represent 2^nd^ and 3^rd^ quartiles and whiskers 1^st^ and 4^th^. Dots denote outliers. Asterisks indicate significance (p < 0.05). **Median working times** (B-D) Median working times plotted for individual animals show performance during the last day of a level and the single first trial of the subsequent level.

**Fig 4:**
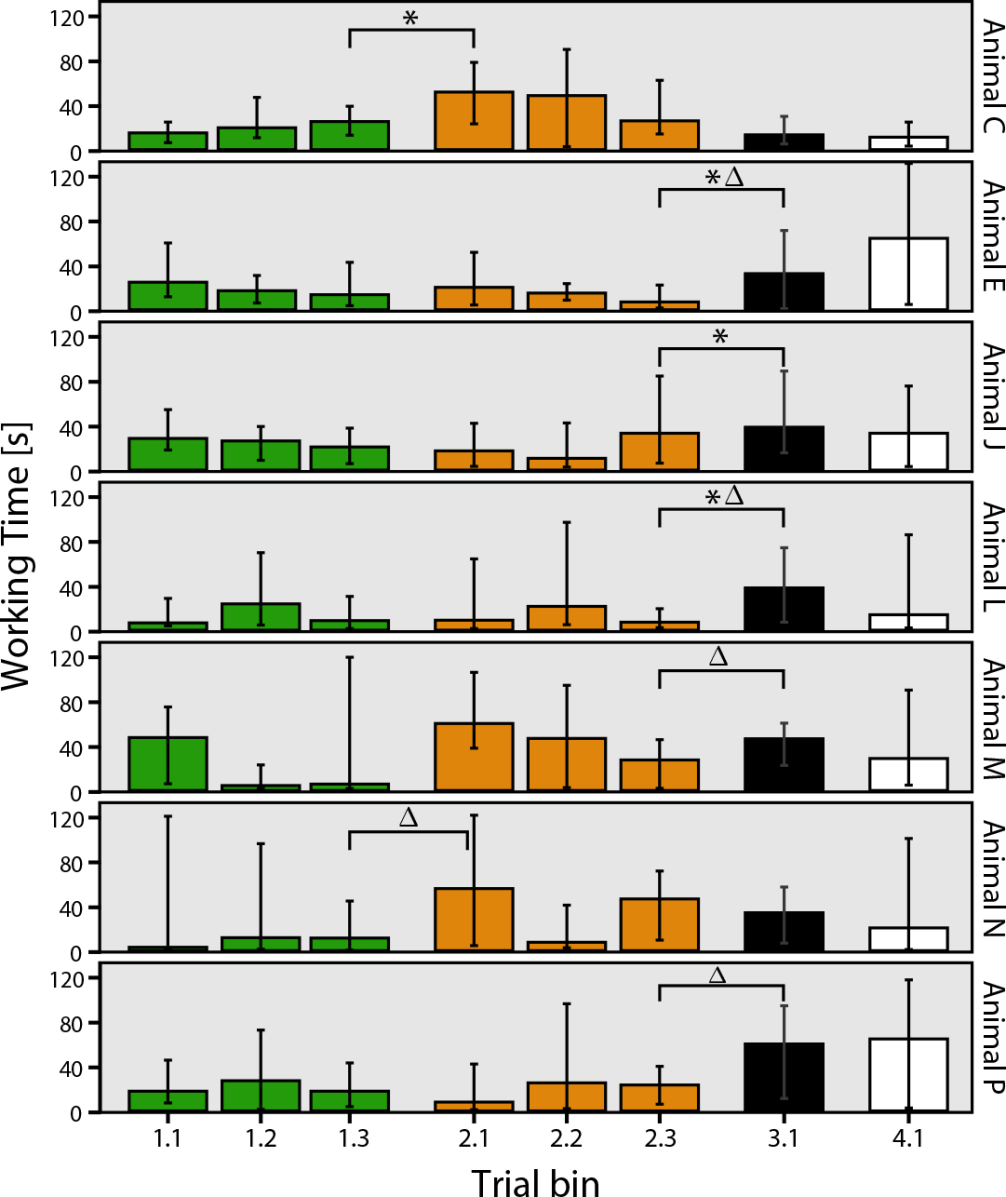
Median working times over trial bins per animal. Green: level 1, introduction of the puzzle-task. Orange: level 2, opaque separator. Black: level 3, reversed container orientation. White: level 4, randomized container orientation. Error bars show 95% CI. Asterisk represents statistical significance between trial bins with p<0.05; triangle represents significance between single experiment days with p<0.05.

To test and compare performance differences between animals and levels (i.e. how fast an animal retrieved the container), over all working times were equally split into three trial bins per level for level 1 and level 2 (Fig 3A). While overall there was no significant difference in working times between level 1 and 2, a test on the single trial bins 1.3 and 2.1, i.e. the last trial bin in level 1 versus the first trial bin in level 2, showed a significant increase in working times for all animals combined (Wilcoxon signed-rank test; z = 3.018, p = 0.003; Fig 3A). However, when analyzing the results of each animal individually, only Animal N had a significant increase in working time between these two trial bins (z = 2.1, p = 0.036; see Fig 4).

To investigate if animals adapted faster to the task alterations than detectable in averaged trial bins, median working times were also compared between key experiment days (Fig 4) and between the averaged last day of a level and the single first trial of the subsequent level (Fig 3B–3D). Grouped animals showed a significant performance decrease again when the first day of level 2 was compared to the last day of level 1 (z = 2.884, p = 0.004). This was probably mostly driven by Animal C, which was the only animal showing a significant increase in working time when compared between days (z = 2.31, p = 0.021; Fig 4). However, by visual inspection of Fig 3B, all but one animal showed a working time increase during the first trial of level 2, compared to their median working time of the previous day, indicating that these animals were affected by the change of the task in level 2, but adapted quickly throughout the experiment day.

### Level 3: Motor learning

In level 1 and level 2 the orientation of the container and therefore the motor procedure was constant, which allowed the possible facilitation of motor learning effects. Since increased performance or a decrease in time could indicate motor learning during the puzzle task, the regression of working time was analyzed for level 1 and level 2 combined. While working times did not decrease significantly for combined animals (Kendall’s tau *τ*_b_ = 0.024, p = 0.37, N = 639; see Fig 3), a single animal significantly decreased working time during these levels (*τ*_b_ = -0.155, p = 0.019, N = 105; see Fig 4, Animal E).

To further test motor learning effects during the problem solving task, the puzzle was altered by reversing the orientation of the container in level 3. Two animals did not reach success criteria in the first day in level 3 (Fig 2, black circles), and when performance was compared between trial bins 2.3 and 3.1 (z = 2.72, p = 0.007; Fig 3A), grouped animals showed a significant decrease in performance, i.e. an increase in working time. This was evident also in 4 individual animals out of the 7 total; Animal E (z = 2.073, p = 0.038), Animal L (z = 2.24, p = 0.025), Animal M (z = 2.073, p = 0.038) and Animal P (z = 2.31, p = 0.021; Fig 4). Three animals showed significant working time differences when compared between the single experiment days of level 2 and level 3, Animal E (z = 2.31, p = 0.021), Animal J (z = 1.992, p = 0.046) and Animal L (z = 2.24, p = 0.025). Five of the seven animals showed a performance decrease on the first trial of level 3, compared to the median working time of the previous day (Fig 3C). Taken together, the data suggest that the animals were affected by the change of orientation of the container but adapted quickly to the new motoric procedure so that five of the seven animals passed the success criteria during a single experiment day.

### Level 4: Behavioral flexibility

**Fig 5:**
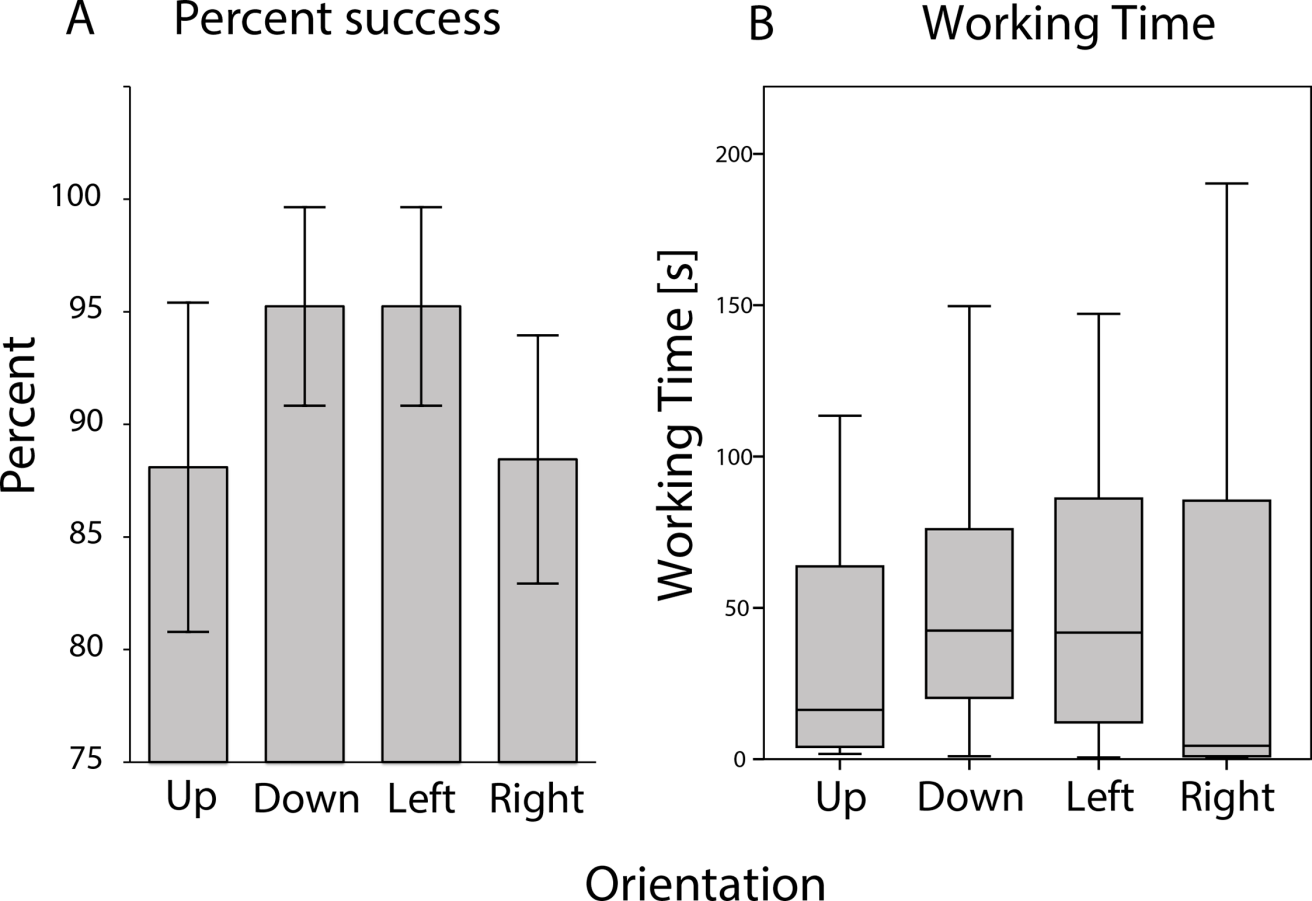
Combined animals during level 4. Orientation of L-shaped box was randomized with equal number of presentations per orientation. (A) Percent success per each of the four orientations. Error bars mark SE. (B) Working time for each orientation.

All animals quickly adapted to changes in the puzzle task. We aimed to test behavioral flexibility by randomizing the orientation of the container in four different positions (level 4). All animals, except Animal M, reached success criteria in the first experiment day (Fig 2, white circles). Overall animals showed a high variance and no significant increase in working time was found between level 3 and level 4. However, an increase in working time was observed for 5 animals between the first trial of level 4 and the last of level 3 (Fig 3D). No significant differences in success rates or working times were found between the four orientations at level 4 (Kruskal-Wallis H test; χ^2^ = 1.415, p = 0.702 and χ^2^ = 5.287, p = 0.152; Fig 5A and 5B), which suggests that the animals used a generalized problem-solving strategy, instead of relying on experience from previous levels.

## Discussion

In order to test for flexible behavior in octopuses, we developed a series of experiments consisting of a learning and a problem solving task. All seven subjects solved the two basic tasks, to open the L-shaped container in level 0 and to pull it through the separator hole in the subsequent puzzle-task levels, and thus demonstrated problem-solving abilities. The animals’ performances differed significantly between level 1, level 2 and level 3, but not level 4 (Fig 3A), suggesting, that they were less affected by the randomized container orientation in level 4 due to a generalized problem solving strategy. Since performances systematically differed between individual animals and tasks, we conclude that the octopuses did not use a trial-and-error strategy throughout the experiment, which would have led to equal performances between tasks, but rather showed individual problem-solving strategies.

### Problem-solving strategies

Innovation, as a form of cognition, stands in relation to other cognitive traits, for example learning, problem-solving and flexible behavior [43]. Performance in these tasks can vary greatly between individuals within a species [44]. We analyzed individual performance differences in order to elucidate if and how octopuses used individual approaches to solve the tasks. The octopuses of the present study generally showed the most variance in performance during the tasks in level 0 and level 1. Level 0 presented a relatively simple problem with a larger fraction being a learning task. Level 1, on the other hand, was more complex: in order to get the object through the tightly fit hole, animals had to overcome their initial instinct to strongly pull on the object and instead, let loose to lever it through. While learning has been shown to be positively related to problem-solving abilities in various birds [45–47], performances were not consistent among octopuses between these levels: One animal (Animal L, see Fig 2) passed this level with the minimum amount of experiment days needed (i.e. three experiment days), showing 100% success rate per day. Animal C on the other hand showed the slowest progress on the task and passed level 1 after 24 experiment days (see Fig 2). On the other hand, Animal C showed over 80% success rate for all experiment days of level 0, while Animal L, with a perfect success score in level 1, needed 16 experimental days in level 0. The individual differences between these two tasks indicate that the classification of an animal into either a “slow” or “fast” learner was not consistent between the levels but rather that solving the learning tasks depended on individual problem-solving strategies. It is interesting to note, that after level 1, success rates stayed relatively constant over 80% and it is plausible to assume that at this level a certain understanding about the general solvability of the problem formed in the animals. It furthermore shows that the animals were motivated to solve the task and that success rates were not dependent on varying motivation.

In level 2, in which by introducing opaque separators eliminated visual information about the container, task-solving times differed between the individual animals. Two animals (Animal C and Animal N) showed an increase in duration to solve the puzzle task. Interestingly, these two animals were the only ones that showed no significant performance decrease in level 3, but instead, showed even faster working times (Fig 4). These results suggest that these animals switched their problem solving strategies from a vision-based to a generalized strategy.

It is unclear if the animals used the visual cues to form a mental representation of the solution to the problem in level 2 or if the presentation of the container itself increased motivation to solve the puzzle task. However, it is likely that the odor cues of the food item inside the unsealed container increased motivation more than the visual cues [38], so that the impact on task duration was probably due to missing visual cues. The other five animals either adapted instantly to the new situation or used different problem solving strategies. Likewise, animals that showed performance loss in level 3 probably adapted to a generalized strategy in level 4, which–with very high working time variances between orientations–resembles what one would expect during trial-and-error.

Behavioral flexibility has been linked to the ability to quickly adjust to novel situations in response to environmental feedback [48, 49]. Griffin and Guez [46] showed that innovation and flexible behavior are not necessarily positively correlated in individual Indian mynas: they showed to be fast learners and problem-solvers, but showed slow adaptation to a reversal task. To test for behavioral flexibility in octopuses, the orientation of the container was reversed in level 3, which led to fast adaptation within the first experiment day and probably the first few trials, however, performance was significantly affected. This resulted in an overall successful experiment day (except animal J; Fig 2) but significant working time increases (Fig 3A) and might reflect the adaptation to a more flexible strategy. Furthermore, in level 4, in which the four different orientations were randomized for every trial, all animals except animal M passed success criteria after the first experiment day and decreased median task duration (but note variance; Fig 3A), which shows that the animals developed a fast and flexible problem solving strategy throughout the previous levels.

Interestingly, in the comparison of the four orientations of the container in level 4, the previously trained orientations ‘up’ and ‘down’ were not necessarily the most successful or the fastest, which hints towards a more generalized puzzle solving strategy, which did not rely on previous knowledge of the orientations. A generalized and hence flexible strategy might consist of motoric elements (e.g. trying to leverage the container in all directions) and intellectual elements (e.g. knowledge about the general shape of the container or mental rotation). A trial-and-error strategy would grant the highest success rate during the constantly changing and unpredictable task, while different strategies might have been advantageous in earlier stages. It is also possible that the animals used sensory cues about the orientation of the containers (e.g. due to slight tilts of the container), which they did not use in earlier levels. At least the common trend towards very short task durations for the ‘right’ orientation would support the use of such strategy. However, although octopuses are able to visually discriminate shapes and to some extent shape rotation [50], tactile shape discrimination seems to be far more complicated. While they are able to discriminate between different surface profiles, orientation and object shapes were indistinguishable [51, 52]. The data on the mechanics of visual shape discrimination remain inconclusive [50]. Taken together, it is very likely that animals resolved to a trial-and-error strategy and it is yet unknown if octopuses have mental representations of objects and if they, with better methodological approaches, would be able to learn to discriminate between different orientations or mirror images, as was shown in e.g. pigeons [53].

Another possible explanation and a potential avenue for further testing, is that performance differences could be due to individual personalities or ‘cognitive styles’ which shaped the animals’ problem-solving strategies (e.g. boldness, activity levels or neophilia) [54]. Personality traits have been shown before in octopuses [55, 56] and while individual personality traits have not been tested during this experiment, some of the results may be explainable by personality differences: While an aggressive personality might have been advantageous in the container-opening task of level 0, it would have been a disadvantage in level 1, which required a more delicate approach. However, the present number of animals is far too small to make any conclusive statement personal traits.

### Motor learning

While solving a problem by insight is an ideal display of cognitive abilities, it is a rarely observed phenomenon in non-primate animals, [57] and at least some knowledge about a certain problem is necessary. A more commonly observed solution to a complex problem is a trial-and-error-approach. One could argue that after repeated presentations to a physical problem, motor learning effects contribute to the solution of a principally similar problem. New Caledonian crows, for example, that were familiar with just the use of short sticks, used the poking-skill to overcome similar problems in a serial, three-step modified trap-tube problem [58].

It has been argued before that motor stereotypy, i.e. the tendency to produce only a narrow range of motor actions, limits the chance to produce high cognitive abilities [59]. It is thought that high motor plasticity and the ability to express novel behaviours during novel situations, is central to innovation [43, 59]. Indeed it has been shown that greater motor diversity can be a predictor for higher problem-soling abilities [60–62]. This is particularly interesting in light of the octopuses’ bodily features: It has been shown that there is some separation of labor between the periphery and the central nervous system [63] and it has been hypothesized, that the central nervous system does not use proprioceptive feedback from the arms [5, 64] and furthermore, that there is a conflict of sensory and tactile information [65, 66]. It has therefore been discussed if octopuses are able to use sensory feedback to control their movements [23, 67], however, recent findings suggest that the animals use at least visual feedback to guide their arms [68] and are able to change motor patterns to adapt to complex motor tasks [41]. One of the most important questions that arises from these theories is, how octopuses learn new motor skills, and adapt to a diverse environment. To test for motor learning effects, the orientation of the container was reversed in level 3, while visual information was still limited. Our working hypothesis was that animals that rely on entrenched motoric procedures to solve the task would then experience a change in performance, either in terms of task duration or success rate. Five of the seven animals were significantly affected by the reversed container (Fig 4). Furthermore, these five animals did not show significant differences in level 2, suggesting, that they were using a motor-oriented strategy early in the experiment.

Motor learning should have a measurable effect on performance, leading to higher effectiveness and hence decrease task duration. Therefore, task duration of experiment days of level 1 and level 2 were analyzed. In the two levels, the orientation of the container and hence motor procedure were the same and each animal had a minimum number of 60 presentations and at least six successive experiment days of 80% success at this point. However, only one animal showed a significant decrease in task duration in this period, which also showed significant differences in level 3, both for trial-bin comparison and day-to-day comparison (Fig 4, Animal E). The trial duration during level 1 and 2 did not show a significant trend to shorter task durations for combined animals, which implies that motor learning does not have a major effect on the animals during the experiments and in turn suggests that performance differences of the five animals rely on certain strategies, instead of motor learning. Octopuses use stereotypic and invariant movement patterns during reaching and fetching movements [69, 70], but it could be shown that they are able to adapt their movement patterns during novel situations [41]. While it remains unclear if octopuses can learn new movements or optimize movement patterns in a repeated motor learning task, they are able to adapt their motor programs. This enables them to solve a wide range of motor tasks and hence the ability to solve complex problems, which would fit the prediction of a positive correlation between motor plasticity and cognition [59].

In conclusion, we show that octopuses exhibit flexible behaviors by being able to quickly adapt to a changing problem. Individual analysis of performance revealed different problem-solving approaches, which exceeded simpler learning mechanisms and did not rely on single fixed strategies like trial-and-error or a stimulus-response-association. However, these strategies can be adapted according to the task and the octopuses probably resort to trial-and-error in unpredictable and changing situations. We furthermore present a non-invasive operant conditioning task, proposing a new way of thinking about complex cognitive experiments on octopuses.

## Supporting Information

S1 Data A SPSS.sav file containing the original data. (SAV)

S1 Text This document contains the NC3Rs ARRIVE Guidelines Checklist Richter et al. (DOCX)

S2 Text This document contains the NC3Rs ARRIVE Guidelines Checklist (filled). (PDF)

S1 Video A video clip of an octopus during a successful trail at level 1, the orientation of the L shaped container is “up”. (MOV)

S2 Video A video clip of an octopus during a successful trail at level 3, the orientation of the L shaped container is “down”. (MOV)
