# Supplementary material for: Pull or Push? Octopuses Solve a Puzzle Problem
Source: PLoS One. 2016 Mar 22;11(3):e0152048. doi: 10.1371/journal.pone.0152048 (PMC4803207; doi:10.1371/journal.pone.0152048)

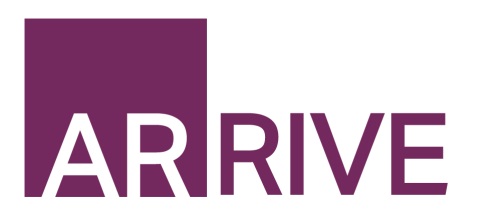


The ARRIVE Guidelines Checklist

Animal Research: Reporting In Vivo Experiments

Carol Kilkenny^1^, William J Browne^2^, Innes C Cuthill^3^, Michael Emerson^4^ and Douglas G Altman^5^

*^1^The National Centre for the Replacement, Refinement and Reduction of Animals in Research, London, UK, ^2^School of Veterinary Science, University of Bristol, Bristol, UK, ^3^School of Biological Sciences, University of Bristol, Bristol, UK, ^4^National Heart and Lung Institute, Imperial College London, UK, ^5^Centre for Statistics in Medicine, University of Oxford, Oxford, UK.*

|  | | ITEM | RECOMMENDATION | Section/ Paragraph |
| --- | --- | --- | --- | --- |
| 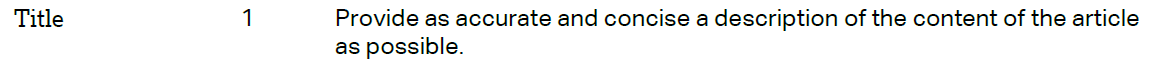 | | | Title |  |
| 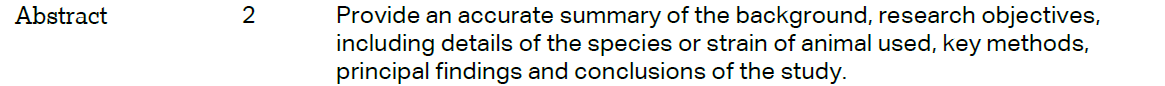 | | | Abstract |  |
| INTRODUCTION | | |  |  |
| 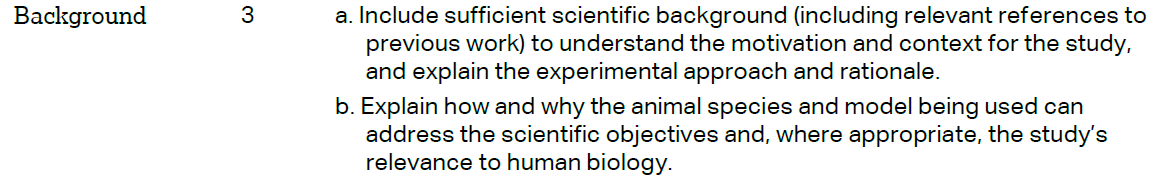 | | | Paragraph 1-2  Paragraph 3-4 |  |
| 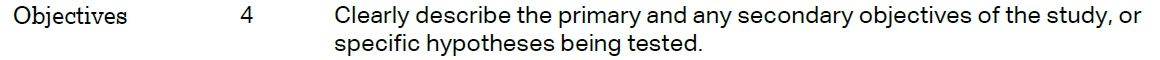 | | | Paragraph 5 |  |
| METHODS | | |  |  |
| 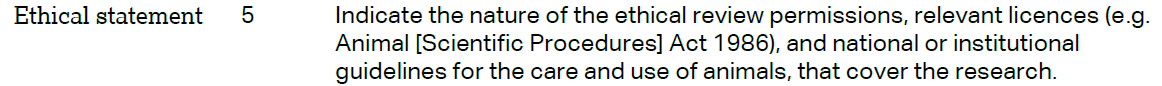 | | | Ethical Statment |  |
| 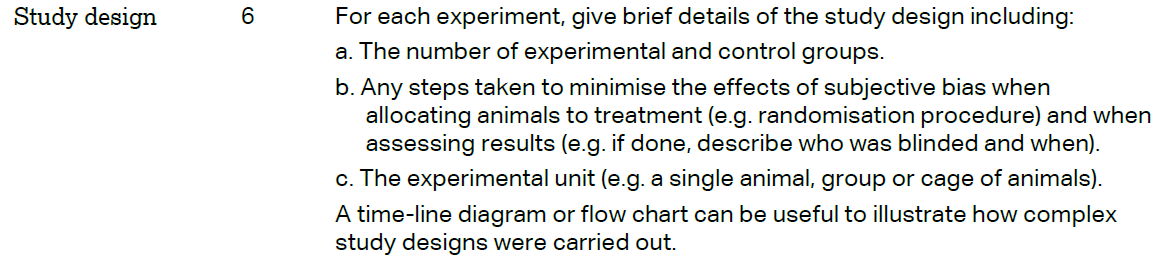 | | | Experiments |  |
| 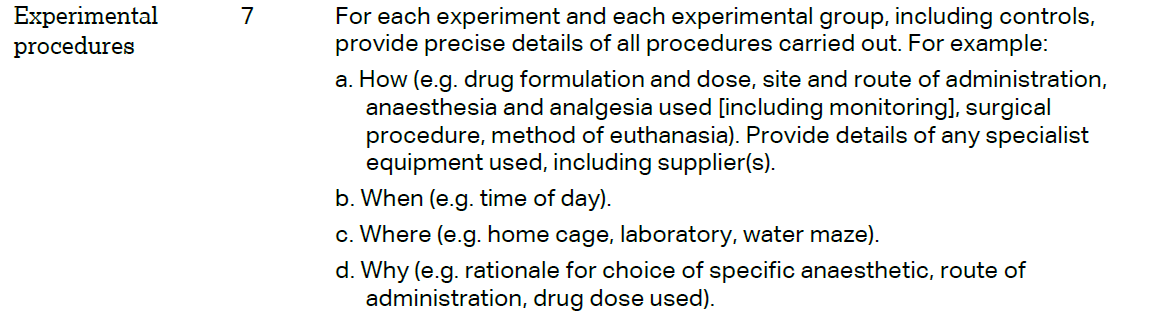 | | | Experiments |  |
| 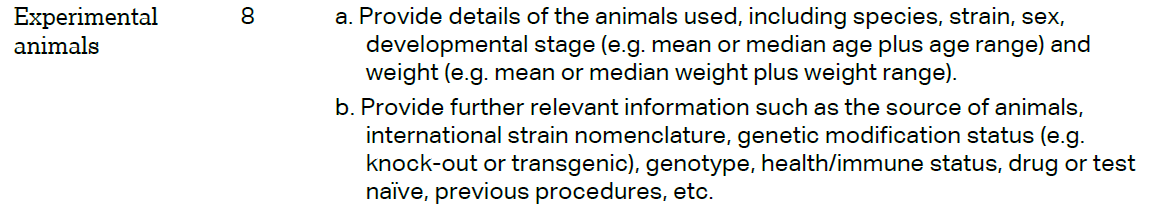 | | | Paragraph 6 |  |

The ARRIVE guidelines. Originally published in *PLoS Biology*, June 2010^1^

| 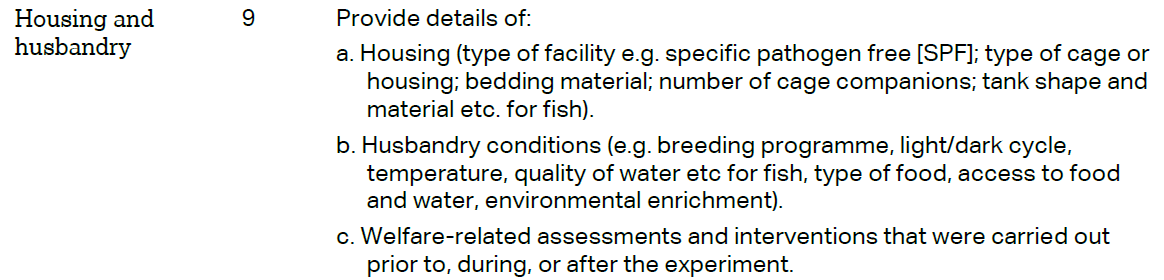 | Paragraph 6 | |
| --- | --- | --- |
| 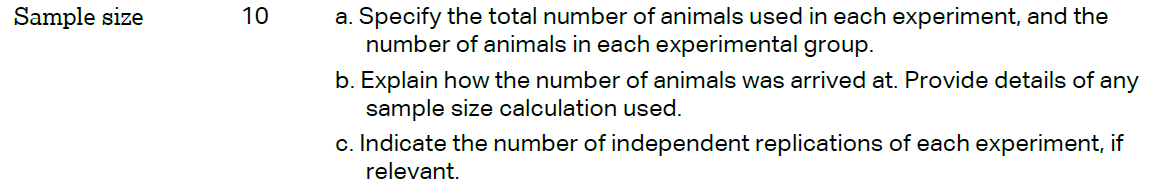 | Paragraph 6 and Analysis | |
| 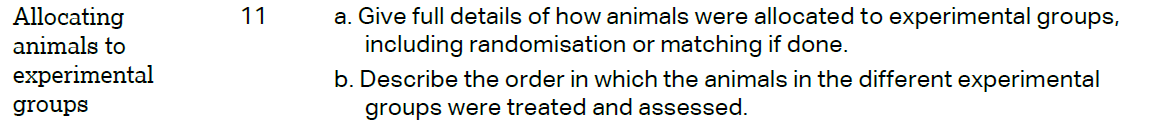 | Experiments | |
| 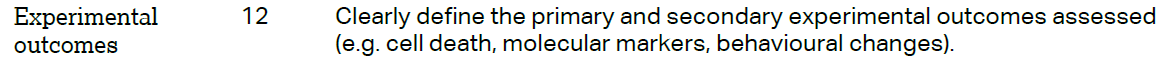 | Experiments | |
| 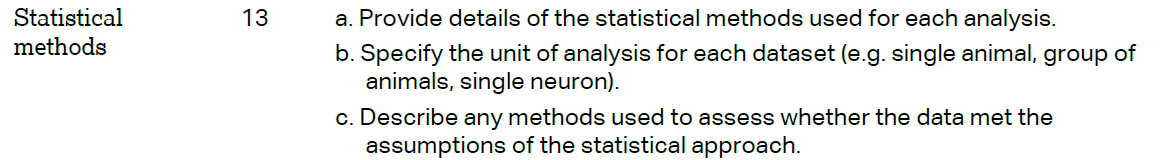 | Experiments | |
| RESULTS |  | |
| 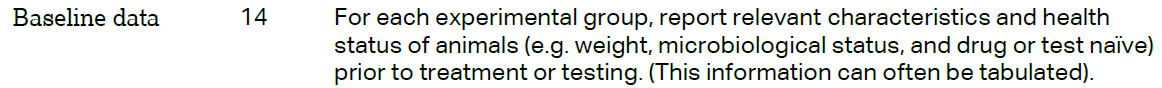 | Not applicable | |
| 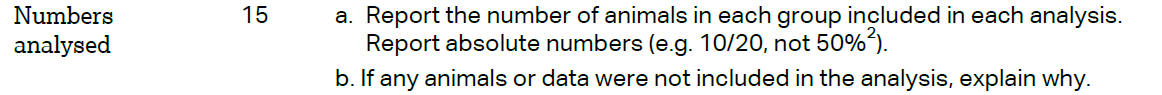 | Analysis | |
| 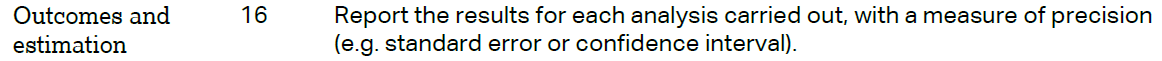 | Results | |
| 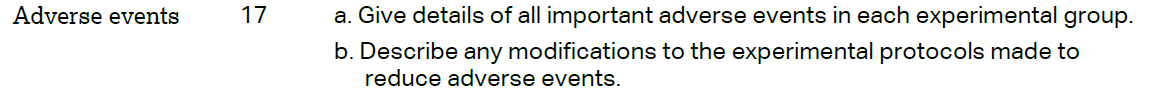 | Not applicable | |
| DISCUSSION |  | |
| 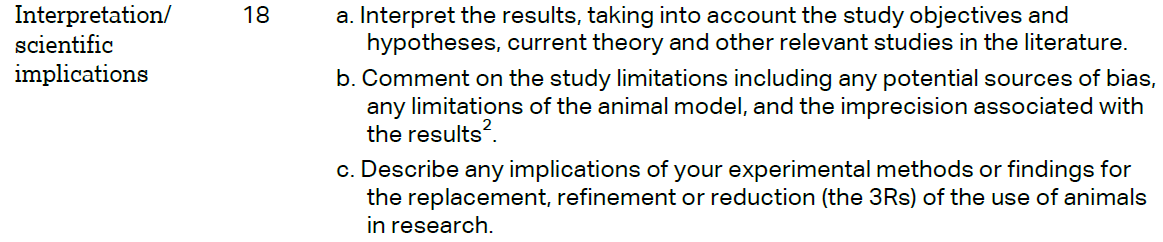 | Discussion | |
| 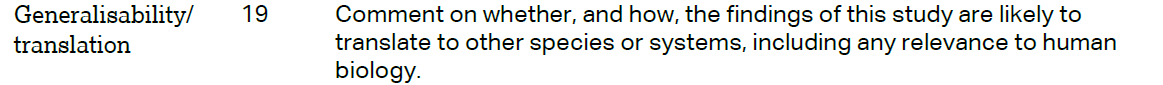 | Discussion | |
| 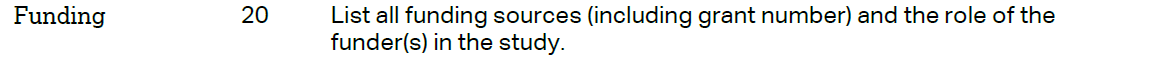 | | Acknowledgements |


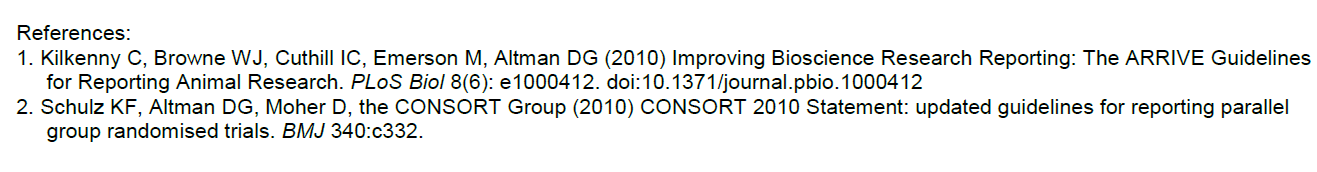

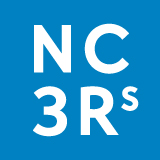

Supplement: S1 Text — (DOCX) [file pone.0152048.s002.docx]
